# Aspirin inhibition and recovery of cyclooxygenase activity and thromboxane biosynthesis in human megakaryocytes: a translational surrogate model

**DOI:** 10.1016/j.jpet.2025.103762

**Published:** 2025-10-28

**Authors:** Zahraa I. Mallah, Giovanna Petrucci, Abeer J. Ayoub, Mohammad A. Farhoud, Joseph G. Jelwan, Sara Lucchisani, Adham K. Fakih, Bassam Badran, Eva Hamade, Carlo Patrono, Bianca Rocca, Aida Habib

**Affiliations:** 1 Department of Biochemistry and Molecular Genetics, American University of Beirut, Beirut, Lebanon; 2 Laboratory of Cancer Biology and Molecular Immunology, Faculty of Sciences I, Lebanese University, Hadath, Beirut, Lebanon; 3 Department of Healthcare Surveillance and Bioethics, Section of Pharmacology, Catholic University School of Medicine, Rome, Italy; 4 Departmental Faculty of Medicine, UniCamillus-Saint Camillus International University of Health and Medical Sciences, Rome, Italy; 5 Department of Biological Sciences, School of Arts and Sciences, Lebanese International University, Bekaa Campus, Bekaa, Lebanon; 6 Department of Translational Medicine and Surgery, Section of General Pathology, Catholic University School of Medicine, Rome, Italy; 7 Center of Excellence on Ageing, CAST, “G. d’Annunzio” University School of Medicine, Chieti, Italy; 8 Department of Medicine and Surgery, LUM University, Casamassima, Bari, Italy; 9 Department of Radiological and Hematological Sciences, Catholic University, Roma, Italy; 10 Department of Basic Medical Sciences, College of Medicine, QU Health, Qatar University, Doha, Qatar; 11 Clinical Translational Science Group, Research and Graduate Studies, QU Health, Qatar University, Doha, Qatar

**Keywords:** Megakaryocytes, Cyclooxygenase-1, Thromboxane, Aspirin, Platelets

## Abstract

Low-dose aspirin irreversibly acetylates cyclooxygenase (COX)-1 in anucleate platelets and progenitor megakaryocytes, permanently suppressing thromboxane (TX)A_2_-dependent platelet activation. Although aspirin pharmacodynamics is well characterized in platelets, the kinetics of COX inhibition and recovery in human megakaryocytes remains poorly defined, due to ethical issues associated with invasive, bone-marrow trephine sampling, and low megakaryocyte yield. We studied aspirin pharmacodynamics in human megakaryocytic cell lines as a reliable and feasible surrogate model. We characterized COX-1 and COX-2 expression and activity in MEG-01 and CHRF-288-11 megakaryocytic cell lines, treated with a range of aspirin concentrations and exposure duration. COX activity was quantified by the production of TXB_2_ from exogenous arachidonic acid. A single 10-*μ*M aspirin exposure suppressed TXB_2_ by 90 ± 2% (MEG-01) and 85 ± 4% (CHRF-288-11), with full recovery within 48–72 hours. Both COX-isozymes were detected by western blot and immunohistochemistry; however, selective COX-1 inhibition by SC-560 reduced TXB_2_ by >75%, whereas COX-2 inhibition by NS-398 had minimal effect. Repeated aspirin exposure every 24 hours produced concentration- and time-dependent TXB_2_ suppression, achieving 89 ± 2% inhibition by day 2 at 1 *μ*M and 73 ± 3% by day 4 at 0.1 *μ*M. TXB_2_ biosynthesis recovered by 86 ± 2% and 99 ± 10% at days 2 and 3, respectively. These findings identify COX-1 as the principal source of TXA_2_ in megakaryocytes and demonstrate that aspirin inhibits megakaryocyte COX-1 time- and dose-dependently, with delayed recovery likely reflecting *de novo* synthesis of COX-1 protein, thereby providing mechanistic insight into the sustained antiplatelet effect of low-dose aspirin in humans.

**Significance Statement:**

In human megakaryocyte cell lines, once-daily aspirin treatment at low-concentration range time-dependently inhibits COX-1 with delayed recovery after aspirin withdrawal. This closely mimics the kinetics of platelets, supporting the translational utility of the megakaryocyte-based surrogate model.

## Graphical abstract

**Figure:**
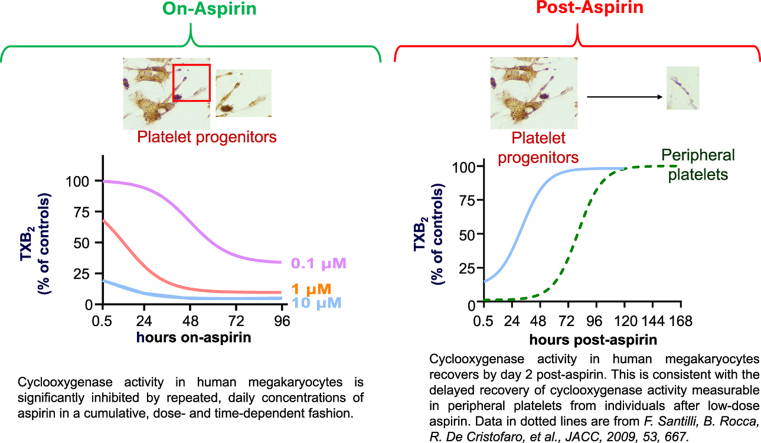

## 1 Introduction

Low-dose aspirin, alone or in association with other antithrombotic drugs, remains central for the prevention and treatment of atherothrombosis.^1^ Prostaglandin (PG)G/H-synthase-1 and -2 isozymes (colloquially referred to as cyclooxygenase [COX]-1 and COX-2) along with the different terminal synthases, catalyze arachidonic acid (AA) metabolism into biologically active prostanoids, such as thromboxane (TX)A_2_, PGD_2_, PGE_2_, PGF_2*α*_, and PGI_2_, depending on the cell-specific enzymatic equipment.^2^ TXA_2_, the main prostanoid synthesized by platelets, has pro-aggregatory and vaso-constrictive properties.^2^ Aspirin permanently acetylates a critical serine residue (Ser-529 in human COX-1, Ser-516 in human COX-2) located at the apex of the COX channel.^2^ This results in irreversible inhibition of prostanoid biosynthesis, which lasts for the entire lifespan of the platelets.^3^ Because TXA_2_ is chemically unstable and short-lived, its platelet production can be assessed by measuring a chemically stable but inactive hydrolysis product, TXB_2_, after whole blood clotting.^4^

In individuals treated with once-daily low-dose aspirin, suppression of platelet TXA_2_ biosynthesis persists for up to 24–48 hours after drug withdrawal, with a lag in recovery despite aspirin’s short half-life in the human circulation (15–20 min).^4^, ^5^, ^6^ This delayed recovery has been attributed to the acetylation and inactivation of COX-1 in bone-marrow platelet progenitors, ie, the mature megakaryocytes (MK), pre- and proplatelets. Most likely, platelets released from bone-marrow MKs during the first 2 days after aspirin withdrawal still contain inactivated COX-1, explaining the 2-day lag in recovery of platelet COX-1 activity,^3^^,^^7^, ^8^, ^9^ which may delay the release in the systemic blood of platelets with unacetylated protein.^4^ Acetylation of COX-1 in the platelet progenitors within the bone marrow has been hypothesized as a major mechanism contributing not only to the persistent TXA_2_ inhibition of blood platelets during the 24-hour dosing interval but also to the 2-day lag in recovery after drug withdrawal,^6^ an hypothesis supported by in silico modeling of the antiplatelet pharmacodynamics of low-dose aspirin. Although the effect of aspirin has been extensively studied in peripheral platelets, its effect on human MKs has only been inferred from measurements of the recovery kinetics of COX-1 activity in peripheral platelets and not directly investigated. Pharmacodynamic studies of primary human MKs resident in the bone marrow are unethical and unfeasible due to the need for an invasive and painful trephine aspiration to collect these cells, considering that MK represent only about 0.1% of nucleated cells under normal conditions. Although MK lineages of human origin are available, in vitro kinetic studies of the inhibition of COX activity by aspirin have not yet been performed. In the present study, we assessed the kinetics of COX activity at different concentrations of aspirin and upon repeated drug exposure in 2 well established human MK cell lines. In particular, we simulated the effects of single and repeated daily dosing and characterized the differential role of COX-isozymes in residual TXA_2_ biosynthesis. Our findings identify COX-1 as the principal source of TXA_2_ in MKs and demonstrate that aspirin inhibits megakaryocyte COX-1 in a time- and dose-dependent manner, with delayed recovery likely reflecting de novo synthesis of COX-1 protein, thereby providing mechanistic insight into the sustained antiplatelet effect of low-dose aspirin in humans.

## 2 Materials And Methods

### 2.1 Reagents

MEG-01 cell line (CRL-2021) was obtained from ATCC^10^ and CHRF-288-11 cells kindly provided by Jean-Philippe Rosa (INSERM U1176).^11^ AA (90010), SC-560 (70340), NS-398 (70590), and TXB_2_-tracer (10005064) for ELISA were purchased from Cayman Chemicals Co. Cell culture media (RPMI-1640, R8758), aspirin (A5376), and mouse *β*-actin antibody (A5441) were from Merck Life Science. RT-PCR reagents including iScript cDNA Synthesis Kit (1708891) and iTaq Universal SYBR Green Supermix (1725121) and electrophoresis, protein quantification, and western blot reagents were from Bio-Rad Laboratories. For immunocytochemistry, EnVision Detection Systems Peroxidase/DAB, Rabbit/Mouse, and HRP (K5007) were from Agilent DAKO Technologies.

### 2.2 Cell culture and treatment

MEG-01 and CHRF-288-11 cells were cultured in RPMI-1640 media supplemented with 50 U/mL penicillin, 50 *μ*g/mL of streptomycin, and 10% or 20% fetal bovine serum (FBS), respectively, in a 5% CO_2_ humidified incubator at 37 °C. Cells were maintained at an approximate density of 0.2–1 × 10^6^ cells/mL, and cell count and viability were assessed using a trypan blue exclusion dye (Merck Life Science T8154). Cells were plated at 2 × 10^5^ cells/mL in 12-well culture plates and pretreated with aspirin (0.1, 1, or 10 *μ*M) for 30 min, then washed. Culture media containing 0.1% ethanol was used as a vehicle. One fraction of the cells was tested for COX activity at day 0 to determine the percentage of baseline inhibition. The rest of the cells were allowed to grow in separate wells for up to 5 days to test COX activity daily. Cells were counted prior to COX activity determination. In other experiments, aspirin was added daily to the cells. CHRF-288-11 cells were established to exhibit markers of MKs and platelets, such as platelet peroxidase, platelet factor 4, platelet Ca^++^-adenosine triphosphatase (ATPase), glycoprotein llb-llla, factor VIII, and the platelet-specific myosin MY7 and MY9, and did not express the erythroid, myeloid nor T, and/or B cell markers.^11^ MEG-01 were established from a blast crisis of Philadelphia chromosome-positive chronic myelogenous leukemia, and they display a similar phenotype as CHRF cells, with a slightly longer replication time.^10^ We further tested the differentiation capacity of the MEG-01 by 50 nM phorbol 12-myristate 13-acetate (PMA) (Merck Life Science P8139) for 4 days and the MEG-01 cells showed a typical morphological transition toward mature MKs (enlarged size and cytoplasmic extensions), consistent with the MK-committed origin of the cell lines (Figure S1).

### 2.3 Immunoblotting and immunocytochemistry of COX-1 and COX-2

Twenty micrograms of total protein was used for western blots. Briefly, CHRF-288-11 and MEG-01 cells were lysed with a protein lysis buffer containing Nonidet-P40 and protease inhibitors (1 mM phenyl-methyl-sulfonyl-fluoride, 1 mM benzamidine, 2 *μ*g/ml aprotinin, 10 mM sodium fluoride, 2 mM sodium orthovanadate, 1 mM sodium pyrophosphate, and 2 *μ*g/mL leupeptin).^12^ The primary antibodies were characterized as previously described^13^ for COX-1, mouse monoclonal antibody anti-COX-1 (clone COX-1-11, 1/1000); for COX-2, mouse monoclonal antibody anti-COX-2 (clone COX-2 29, 1/2000) and mouse *β*-actin (dilution 1/5000). Clarity western ECL substrate (Bio-Rad Laboratories 170-5061) was used according to the manufacturer’s instructions to reveal positive bands visualized using ChemiDoc (Bio-Rad Laboratories).

Immunocytochemistry (ICC) of MEG-01 cells was performed using anti-COX-1 (final dilution, 1:400) and anti-COX-2 (1:800) noncommercial polyclonal antibodies^14^ as previously described.^13^^,^^15^ the antibodies were revealed using the EnVision Detection Systems Peroxidase/DAB according to the manufacturer’s instructions (Agilent DAKO Technologies). Normal rabbit serum (Vector Laboratories) was used as a negative control.

### 2.4 Cyclooxygenase activity

To determine COX activity, cells were washed twice with PBS before adding 10 *μ*M AA in Hanks buffer, pH 7.4, containing 1 mg/mL BSA for 30 min as previously described.^12^ Cells were collected on ice and centrifuged for 15 min at 300 g at 4 ˚C. Supernatants were stored at −70 ˚C. We investigated MK biosynthesis of TXA_2_ by measuring TXB_2_. TXA_2_ is the main prostanoid produced by platelets and their progenitors.^16^ TXB_2_ was measured in the cell supernatants by enzyme immunoassay as previously described, using a specific rabbit anti-TXB_2_ serum.^17^ In some experiments, cells were pretreated with 10 *μ*M SC-560, a selective COX-1 inhibitor, 1 *μ*M NS-398, a selective COX-2 inhibitor, or DMSO (0.1%; v,v) 30 min before adding AA and measuring TXB_2_. Drug concentrations were selected as previously described.^18^^,^^19^

### 2.5 Reverse transcriptase-PCR

Cells were treated with a daily dose of 10 *μ*M aspirin or vehicle for 3 days, total RNA was extracted, and 1 *μ*g was reverse-transcribed. RT-qPCR was performed on the CFX384 cycler (Bio-Rad Laboratories) using the iTaq Universal SYBR Green Supermix as previously described.^12^ Gene expression of COX-1 (*PTGS1)*, COX-2 (*PTGS2)*, TXA_2_ receptor (*TXA2R)*, and TX-synthase *(TXAS)* was evaluated. Primers used in this study are reported in Table S1 and were obtained from Eurofins Genomics. The results were calculated using the 2-^ΔΔCt^ method normalized against the 18S housekeeping gene.

### 2.6 Data analysis

TXB_2_ concentrations measured as pg/mL were corrected for the number of cells per well (ng/million cells). The percentage of TXB_2_ formed in aspirin-treated cells *vs* vehicle-treated cells (control) was determined based on data from 3 to 4 independent experiments, each performed in 3–4 replicates, and is expressed as mean ± standard error of the mean (SEM). Statistical analysis was conducted using GraphPad Prism 10 (GraphPad Software) using unpaired *t*-test or 1-way ANOVA (with Tukey post hoc analysis). *P* values < .05 were considered statistically significant.

## 3 Results

### 3.1 COX-isozyme expression and activity

**Fig. 1:**
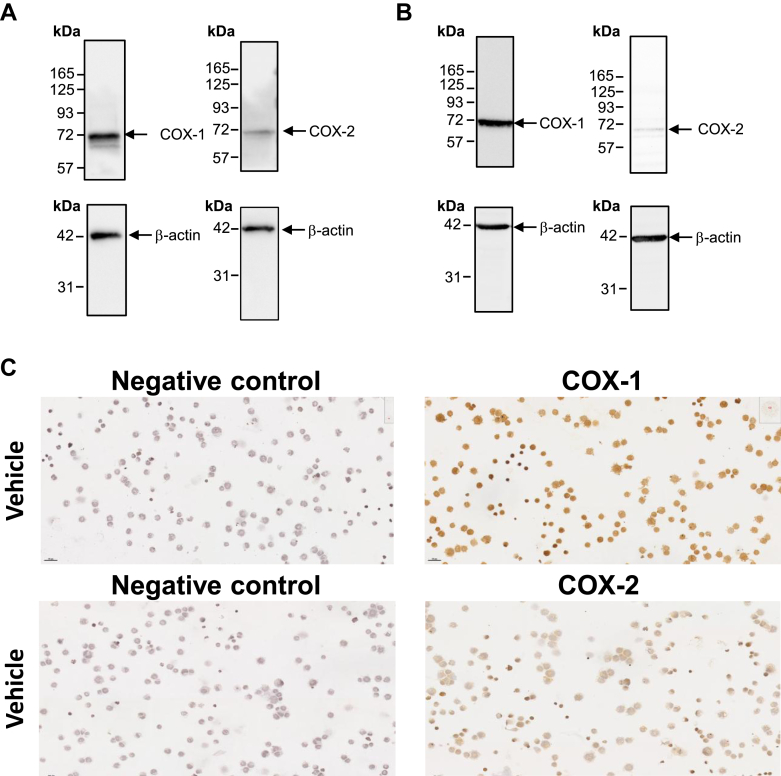
Expression of COX-1 and -2 in MEG-01 and CHRF-288-11. (A) Immunoblot analysis of COX-1 and COX-2 expression in CHRF-288-11 cells. (B) Immunoblot analysis of COX-1 and COX-2 expression in MEG-01 cells. Two × 10^6^ cells were lysed, and 20 *μ*g of total proteins were subjected to 9% SDS-polyacrylamide gel. COX-1, COX-2, and *β*-actin were developed on the same blot (*n* = 3). (C) Immunocytochemistry of MEG-01 cells using selective anti-COX-1 and COX-2 antibodies. Representative images of immunostaining of COX-1 or COX-2 are shown; slides were analyzed by light microscopy using the PhenoImager HT 2.0 workstation system (Akoya Biosciences) at main magnification ×20. COX, cyclooxygenase.

COX-1 and COX-2 proteins were detected by immunoblotting in CHRF-288-11 (Fig. 1A) and MEG-01 cells (Fig. 1B) confirming previous data obtained in MK cell lines, undifferentiated CD34^+^ stem/progenitor cells and thrombopoietin-CD34^+^ differentiated cells.^16^^,^^20^ The expression of mRNA for COX-1 and COX-2, TXA_2_ receptor (TP), and TX-synthase was confirmed by RT-PCR (Table S2). Both COX-isozymes were detectable in MEG-01 by ICC (Fig. 1C**)**.^20^

In addition to COX-isozyme expression in the 2 MK cell lines, COX activity was studied upon AA loading, to reflect maximal biosynthetic capacity.^21^ TXB_2_ biosynthesis was 3.8 ± 0.47 and 2.15 ± 0.55 ng/10^6^ cells in MEG-01 (mean ± SEM; *n* = 23 experiments performed in triplicate) and CHRF-288-11 (*n* = 10 experiments in triplicate), respectively.

### 3.2 Dose-dependent effects of a single aspirin concentration and selective COX-isozyme inhibitor treatment

**Fig. 2:**
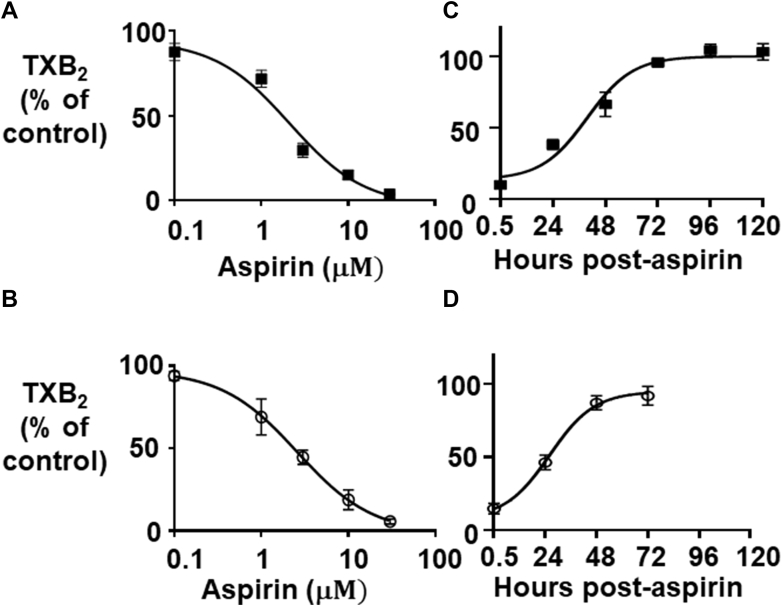
Effect of aspirin on TXB_2_ biosynthesis and kinetics of COX activity recovery in MEG-01 and CHRF-288-11 cells. (A, B) MK cells were treated with different concentrations of aspirin (0.1, 1, 3, 10, and 30 *μ*M) for 30 min, followed by TXB_2_ measurement. The percentage of TXB_2_ in aspirin-treated cells *vs* vehicle-treated cells as control is plotted. (A) MEG-01 and (B) CHRF-288-11. Results are expressed as a mean ± SEM of experiments performed in triplicates in MEG-01 (*n* = 7) and CHRF-288-11 (*n* = 3), respectively. IC_50_ was determined by GraphPad Prism and calculated as 2.4 ± 0.5 and 2.8 ± 0.6 *μ*M (mean ± SEM.) for MEG-01 and CHRF-288-11, respectively. (C, D) MK cell lines were treated with 10 *μ*M aspirin or vehicle (0.1% ethanol, v/v) for 30 min and COX activity was determined. Washed cells were allowed to recover for 24, 48, 72, 96 and 120 hours. TXB_2_ levels in (C) MEG-01 and (D) CHRF-288-11 were measured after AA loading and expressed as % TXB_2_ of aspirin vs control (vehicle-treated cells). Results are expressed as a mean± SEM. of separate experiments for MEG-01 (*n* = 10) and for CHRF-288-11 (*n* = 8), performed each in triplicate. AA, arachiconic acid; COX, cyclooxygenase; TX, thromboxane.

We determined the dose dependence of TXA_2_ inhibition by aspirin. Aspirin concentration-dependently lowered TXB_2_ levels in MEG-01 (Fig. 2A) and CHRF-288-11 cells (Fig. 2B) with IC_50_ values of 2.4 ± 0.5 and 2.8 ± 0.6 *μ*M, respectively. Thus, in subsequent experiments, 10 *μ*M aspirin was used to achieve full suppression of TXB_2_ biosynthesis.

To investigate the recovery kinetics of the TXA_2_ biosynthetic capacity after full inhibition, COX activity was measured daily for up to 120 hours after a single treatment with 10 *μ*M aspirin. TXB_2_ biosynthesis showed substantial recovery by 48 hours and fully recovered at 72 hours. Both cell lines maintained this restored biosynthetic capacity and aspirin treatment showed no toxicity, with 85%–100% viable cells at all-time points compared with controls (Figs. 2C and 2D).

**Fig. 3:**
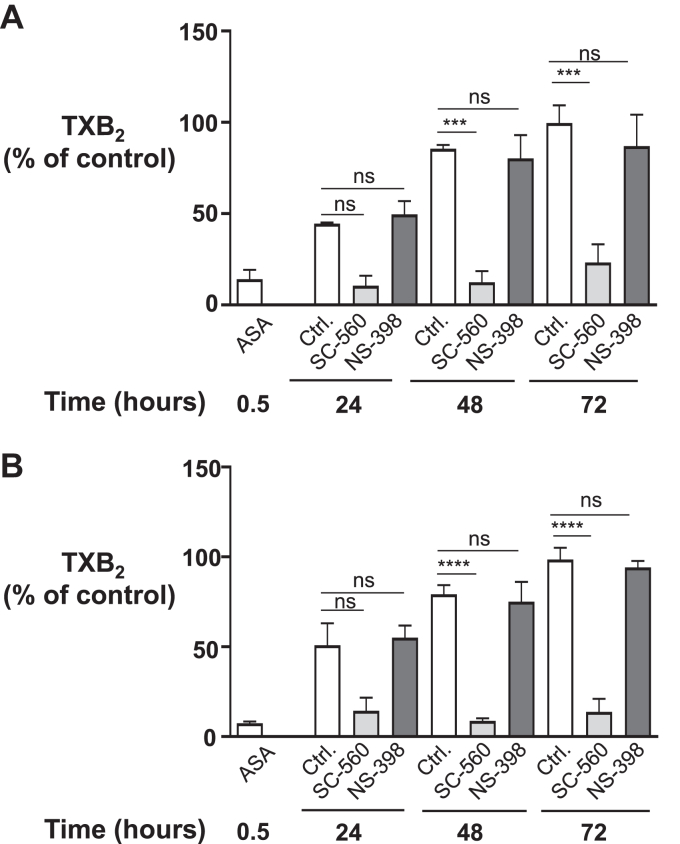
COX isoenzyme contribution in TXB_2_ biosynthesis after treatment with 10 *μ*M aspirin in (A) MEG-01 and (B) CHRF-288-11 cells. All cells were treated with 10 *μ*M aspirin for 30 min, and basal COX activity was determined (labelled ASA). Cells were next washed and plated for each time point (24, 48, and 72 hours), treated with either DMSO (0.1%; v/ v) (labeled Ctrl.), 10 *μ*M SC-560 or 1 *μ*M NS-398 for 30 min, before the addition of 10 *μ*M AA (see the Methods section). TXB_2_ levels were measured as ng/million cells and expressed as % of control. Results are expressed as a mean ± SEM. of three experiments performed in triplicate for both MK cell lines, and statistical analysis was performed using 1-way ANOVA followed by Tukey test, comparing each inhibitor vs control at each time point. ∗∗∗*P* < .001; ∗∗∗∗*P* < .0001. AA, arachidonic acid; ASA, aspirin; Ctrl., Control; ns, nonsignificant; TX, thromboxane.

Because both COX-isozymes are expressed by MEG-01 and CHRF-288-11 cell lines, we investigated their relative contribution to TXA_2_ biosynthesis during the recovery phase following a single aspirin treatment, using selective COX-1 and COX-2 inhibitors (SC-560 and NS-398, respectively). On day 1, cells were treated with 10 *μ*M aspirin or vehicle, then washed and maintained in culture for a total of 72 hours. Each subsequent day, cells were incubated with 10 *μ*M SC-560, 1 *μ*M NS-398, or vehicle, and TXB_2_ was measured. In vehicle-treated MEG-01, TXB_2_ biosynthesis recovered by approximately 86 ± 2% and 99 ± 10% at 48 and 72 hours, respectively, whereas the recovery was significantly lowered by SC-560, ie, 12 ± 6% and 23 ± 10%, at 48 and 72 hours, respectively (Fig. 3A). Similar results were obtained in SC-560-treated CHRF-288-11 cells, with recoveries of 9 ± 1% and 14 ±7 % at 48 and 72 hours, respectively (Fig. 3B). At variance with SC-560 treatment, TXB_2_ recovery at 48 and 72 hours was minimally affected by NS-398 (80 ± 13% and 87± 17% in MEG-01; 75 ± 11% and 94 ± 4% in CHRF-288-11, respectively). These results suggest a prevailing COX-1 involvement in TXA_2_ biosynthesis during the recovery phase following aspirin withdrawal in both MK cell lines.

### 3.3 Effects of daily aspirin treatment on TXA_2_ biosynthesis

**Fig. 4:**
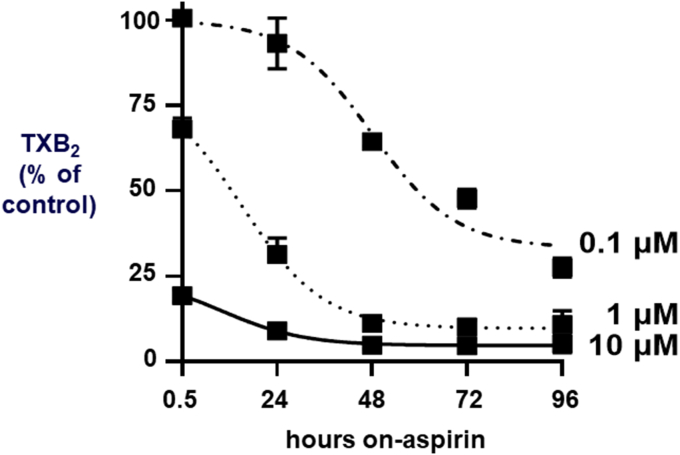
Effect of daily treatment with aspirin on TXB_2_ biosynthesis in MEG-01. Cells were treated at day 0, washed, and treated daily with 10, 1, or 0.1 *μ*M aspirin. COX activity was assessed each day by TXB_2_ expressed in ng/million cells and represented as a percentage of control (mean ± SEM, *n* = 3–4, performed in triplicate). Statistical analysis was performed using one-way ANOVA followed by Tukey test, ∗∗∗∗*P* < .0001. TX, thromboxane.

We next explored the effects of repeated once-daily aspirin treatment at lower concentrations on the kinetics of COX inhibition. As control for maximal inhibition, both MK cell lines were treated with vehicle or 10 *μ*M aspirin once-daily for up to 96 hours. COX activity at each daily time point showed a nearly complete and steady inhibition (Fig. 4 and Fig. S2).

Based on the concentration-response curve (Fig. 2), we expected that a single treatment with 0.1 and 1 *μ*M aspirin would produce 12 ± 5% and 28 ± 5% TXB_2_ inhibition, respectively, in the MEG-01 cells. Thus, we evaluated whether these low concentrations of aspirin would achieve a time-dependent increasing level of inhibition upon repeated, once-daily treatments, as a function of cumulative acetylation of COX-1. MEG-01 cells were treated multiple times with 0.1, 1 *μ*M, 10 *μ*M aspirin, or vehicle and tested for TXA_2_ inhibition. After 48 hours, 1 *μ*M aspirin achieved 89 ± 2% inhibition, which was similar to the effect of 10 *μ*M (95 ± 1%; *P* = ns) (Fig. 4), and remained steady thereafter. The repeated once-daily treatment with 0.1 *μ*M aspirin achieved a progressively increasing inhibition, which was maximal by 96 hours (73 ± 3%), but remained significantly lower than the effect of 10 *μ*M (Fig. 4).

## 4 Discussion

Despite extensive research on low-dose aspirin in cardiovascular treatment and prevention,^1^ the study of its pharmacological effects on bone-marrow MKs has been hampered by limited accessibility of primary MKs from the bone marrow due to ethical, anatomical, and practical reasons, particularly for kinetic studies requiring repeated daily sampling.^22^

The likely involvement of MK inhibition in aspirin antiplatelet pharmacodynamics has been inferred from the 48-hour lag in the recovery of COX activity following aspirin withdrawal, as characterized in blood platelets by measuring the degree of acetylation of the enzyme^3^ and TXB_2_ production ex vivo.^9^ This hypothesis is supported by in silico modeling of the antiplatelet pharmacodynamics of low-dose aspirin,^8^ but a surrogate MK cell model would be required to confirm indirect measurements and in silico data. Thus, to investigate the dose- and time-dependence of the inhibition and recovery of COX activity upon aspirin exposure and following its withdrawal, we used 2 validated and phenotypically well characterized human MK cell lines.^10^^,^^11^ These cell lines have been used extensively to investigate human megakaryopoiesis.^23^^,^^24^

We found that both CHRF-288-11 and MEG-01 cells express COX-isozymes and synthesize TXA_2_ after stimulation with AA at levels comparable to those reported in MKs generated from human CD34^+^ progenitor cells differentiated *in vitro* with thrombopoietin,^16^ supporting their suitability as a surrogate model. Moreover, both cell lines showed similar IC_50_ values for TXA_2_ inhibition by aspirin, with a largely prevalent COX-1-dependent contribution, in line with previous studies.^16^^,^^24^^,^^25^ Interestingly, the aspirin concentrations required for inhibition of COX-1 activity of MK cell lines were in a low-micromolar range compatible with peak plasma levels of acetylsalicylic acid measured in healthy subjects following the oral administration of 100 mg enteric-coated aspirin.^26^

We showed that the full suppression of TXA_2_ biosynthesis was achieved after exposure to a single, high concentration of aspirin, which may mimic the loading dose of aspirin administered in the setting of acute coronary syndromes, followed by a full recovery of COX activity within 48–72 hours. Because platelet COX-1 is irreversibly inactivated by aspirin, the recovery of TXA_2_ production after aspirin withdrawal in humans can only be sustained by de novo synthesis of COX-1 protein in MKs that is transferred to developing platelets which enter the bloodstream upon maturation and detachment from the parent MK. This relatively slow pattern of recovery of COX-1 activity upon aspirin withdrawal from MK cell lines may explain the complete suppression of platelet TXB_2_ production during the 24-hour dosing interval of aspirin administration, despite a 10%–12% release of newly formed platelets. This time frame is consistent with data obtained in rat primary MKs where TXB_2_ synthesis was fully restored within 72 hours^27^ and with the COX-1 protein half-life of 12–24 hours described for fibroblasts and recombinant enzyme,^28^ as well as with the COX-1 mRNA half-life of approximately 15 hours,^29^ which would be needed to resynthesize and replace the permanently inactivated COX-1.^15^^,^^16^ Moreover, Patrignani et al^26^ have previously reported a persistent level of acetylated platelet COX-1 throughout the 24-hour aspirin dosing interval, a finding consistent with the hypothesis that the drug acetylated COX-1 in the bone-marrow MKs.

Another novel finding of our study is that repeated daily exposure of MK cell lines to a low aspirin concentration range, ie, 0.1 and 1 *μ*M, well below the IC_50_, achieved a progressively increasing inhibition of TXA_2_ biosynthesis, which plateaued after 3–4 days in a concentration-dependent manner. This finding is consistent with previous studies in healthy volunteers receiving once-daily low-dose (20–30 mg) aspirin, in whom the degree of platelet COX acetylation and inhibition of TXA_2_ biosynthesis increased in a cumulative fashion over time.^3^^,^^9^

Our study has several limitations. First, immortalized human cell lines may not fully reproduce the phenotype of primary human MKs isolated from the bone marrow or differentiated from CD34^+^ stem cells. However, both MEG-01 and CHRF-288-11 share similar properties with primary MK and have been used extensively to study human MK development and function.^23^^,^^24^^,^^30^^,^^31^ Second, because COX-1 protein is highly stable in most cells including MEG-01 (>60 hours)^20^^,^^32^ and western blot cannot distinguish acetylated from nonacetylated COX-1, measurement of TXB_2_ biosynthesis was assumed to reflect the activity of newly synthesized COX-1 in MK cell lines during the recovery phase. Furthermore, the present study does not address potential pharmacokinetic differences between peripheral plasma and the bone-marrow compartment. Thus, distinct microenvironmental factors in the bone marrow, such as differential perfusion, local metabolism, and cellular uptake, could significantly influence local drug availability and pharmacodynamic effects. We previously developed a novel in silico model describing the key features of aspirin PK/PD in man, namely, its kinetics in the circulation, irreversible inactivation of COX-1 in peripheral platelets and in bone-marrow precursors, and synthesis and replacement of acetylated COX-1.^8^ A model sensitivity analysis performed to identify the main processes influencing COX-1 dynamics and in silico experiments indicated a major role for MKs and platelet turnover in determining the extent and duration of COX-1 inhibition by once-daily low-dose aspirin. In silico simulations also predicted the superiority of reducing the dosing interval vs increasing the once-daily dose in conditions of increased platelet turnover.^7^ These predictions were confirmed by the recent Aspirin Regimens in Essential Thrombocythemia (ARES) trial in patients with essential thrombocythemia, a disorder of increased MK proliferation.^33^

## 5 Conclusion

Within the limitations of a surrogate in vitro model, our study demonstrates that COX-1 activity of human megakaryocytic cell lines is sensitive to low-micromolar aspirin concentrations and displays a slow pattern of recovery following drug withdrawal. These findings provide mechanistic insight into the sustained antiplatelet effect of low-dose aspirin in humans and the nonlinear pattern of recovery of COX-1 activity in blood platelets upon drug discontinuation. A similar surrogate model may be applicable to investigating the contribution of the MK compartment to the antiplatelet pharmacodynamics of irreversible P2Y_12_ blockers.

## Conflict of interest

CP reports personal fees from AbbVie and past grant support (to the Institution) for investigator-initiated research from AIFA (Italian Drug Agency), Bayer, Cancer Research UK, and European Commission; he chaired the Scientific Advisory Board of the International Aspirin Foundation. CP, GP, and BR are currently members of the SPARC Consortium funded by Cancer Research UK (grant agreement PRCNPG-Nov24/100001). All other authors declare no competing financial interests.
