# Supplementary material for: Aspirin inhibition and recovery of cyclooxygenase activity and thromboxane biosynthesis in human megakaryocytes: a translational surrogate model
Source: J Pharmacol Exp Ther. 2025 Oct 28;392(12):103762. doi: 10.1016/j.jpet.2025.103762 (PMC12799507; doi:10.1016/j.jpet.2025.103762)

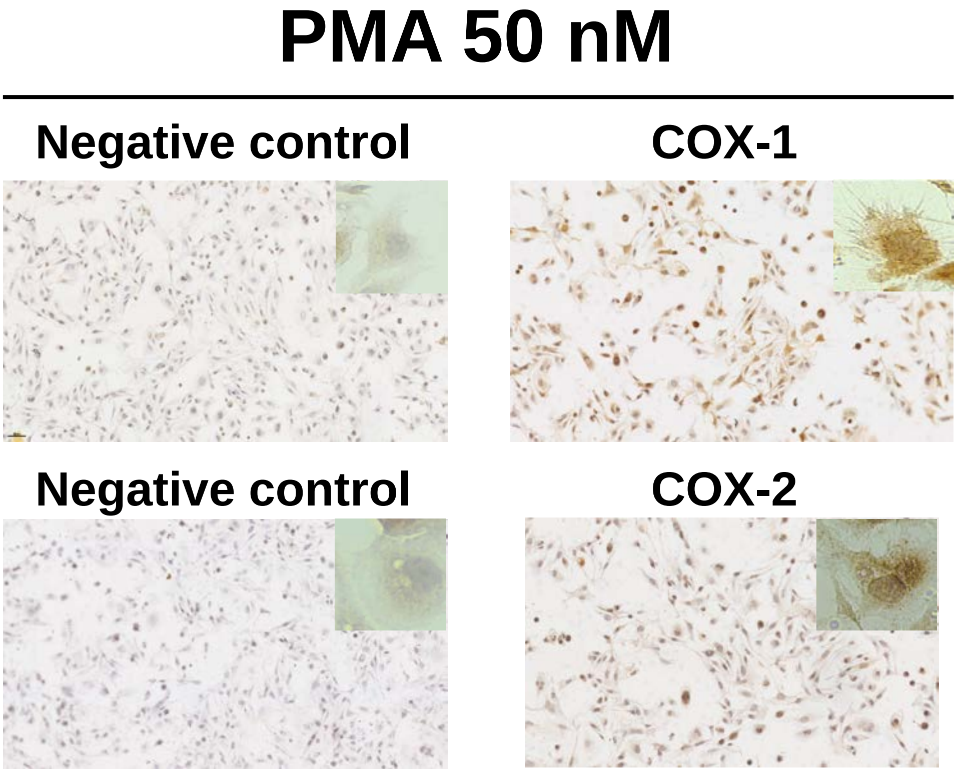

Figure S1: COX-1 and -2 in PMA-treated MEG-01.

MEG-01 cells were treated with 50 nM PMA, and immunocytochemistry was performed using selective anti-COX-1 and COX-2 antibodies. Representative images of immunostaining of COX-1 or COX-2 are shown; Slides were analyzed by light microscopy using the PhenoImager® HT 2.0 workstation system (Akoya Biosciences, Marlborough, MA, USA) at main magnification x20 and a higher magnification of x40 for the inserts, supporting brightfield whole-slide imaging. COX, cyclooxygenase; PMA, phorbol 12-myristate 13-acetate.

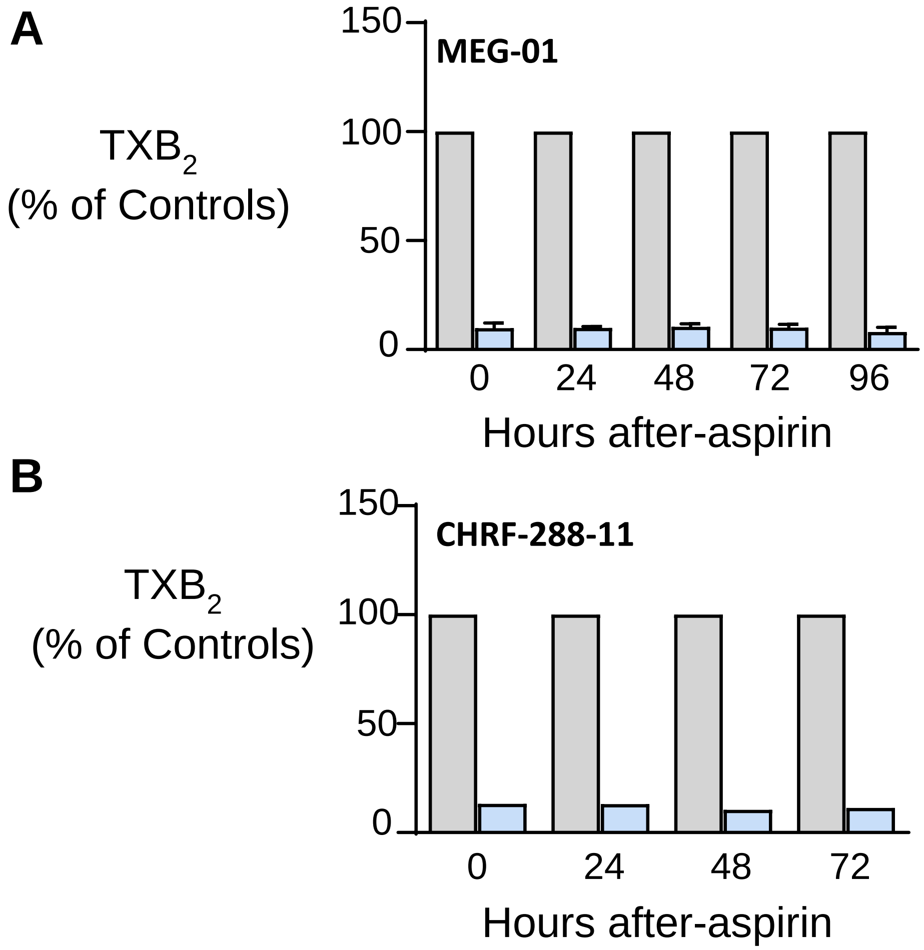

Figure S2: Effect of daily treatment with aspirin (10 µM) on TXB_2 biosynthesis in (A) MEG-01 and (B) CHRF-288-11. Cells were treated on the first day and daily with 10 µM aspirin. Cyclooxygenase activity was assessed each day. TXB_2 levels are expressed in ng/million cells and represented as a percentage of vehicle-treated cells (controls). Results shown are mean ± SEM of 3 experiments for MEG-01 and 2 for CHRF-288-11, respectively, performed in triplicate. TX, thromboxane.

Table S1: List of human primer sequence used in quantitative PCR

Gene name	Forward primer	Reverse primer
RNA18S	5’-AACTTTCGATGGTAGTCGCCGT-3'	5’-TCCTTGGATGTGGTAGCCGTTT-3'
PTGS1	5’-CGCCATGAGCCGGAGTCT-3'	5’- CTGGTGCTGGCATGGATAGT-3’
PTGS2	5’-TGCTGGCAGGGTTGCTGGTGGTA-3'	5’ GGGCTTCAGCATAAAGCGTTTGCGG-3’
TXAS	5’-TTTCTACCTGCAGAGCACGG-3'	5’-TGCTGATGTGGAGTACCATTTC-3’
TBXA2R	5’-GAGGTCTCTGAAGGTGTGCC-3'	5’-CCGTCTCTCCTCCAGGGTAA-3’

Table S2: The cycle threshold of some genes of the prostanoid synthesis and signaling pathway in MEG-01 and CHRF-288-11 cells

	PTGS1	*PTGS2*	*TXAS*	*TBXA2R*	*RNA18S*
MEG-01	21.94	22.47	23.45	27.45	14.43
CHRF-288-11	25.88	24.32	24.42	27.24	15.13
RT[a]	34.5	32	32.6	32.8	26.8

[a]Cycle threshold was reported for samples where no reverse transcriptase was added during the cDNA synthesis and corresponds to negative control.

Figure 1: Expression of COX-1 and -2 in MEG-01 and CHRF-288-11.

(A) Immunoblot analysis of COX-1 and COX-2 expression in CHRF-288-11 cells.

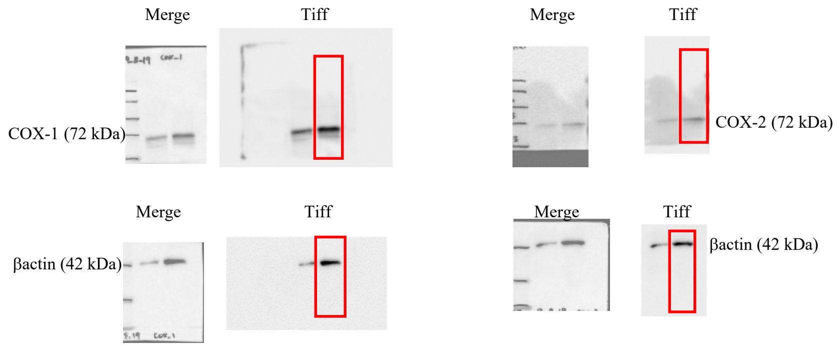

B) Immunoblot analysis of COX-1 and COX-2 expression in MEG-01 cells.

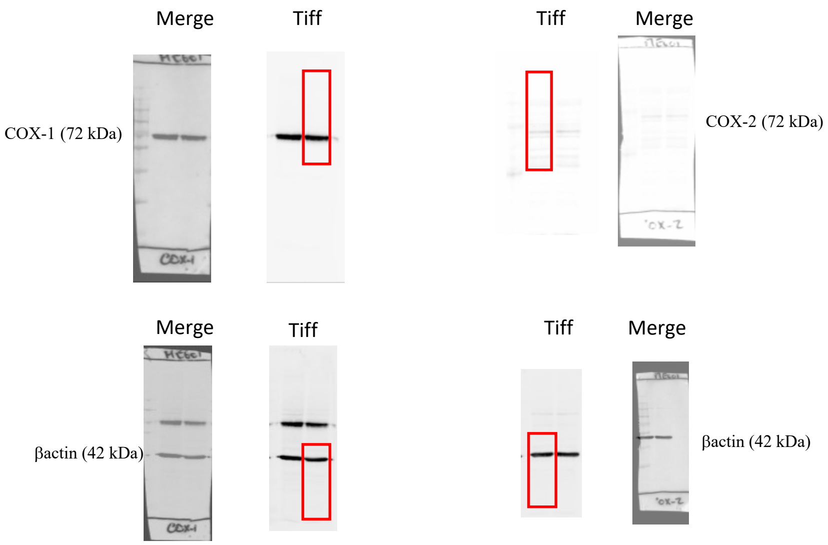

Supplement: Supplementary Figures 1-2 and Supplementary Tables 1-2 [file mmc1.pdf]
